# Exogenous Nitric Oxide and Phosphorus Stress Affect the Mycorrhization, Plant Growth, and Associated Microbes of *Carya illinoinensis* Seedlings Colonized by *Tuber indicum*

**DOI:** 10.3389/fmicb.2019.02634

**Published:** 2019-11-13

**Authors:** Xiaoping Zhang, Xiaolin Li, Chenguang Wu, Lei Ye, Zongjing Kang, Xiaoping Zhang

**Affiliations:** ^1^Department of Microbiology, College of Resources, Sichuan Agricultural University, Chengdu, China; ^2^Soil and Fertilizer Institute, Sichuan Academy of Agricultural Sciences, Chengdu, China

**Keywords:** *Tuber indicum*, ectomycorrhizae, nitric oxide, phosphorus stress, truffle

## Abstract

In the artificial cultivation of truffles, ectomycorrhizal colonization level, host plant quality, and the associated microbes in the rhizosphere soil are vitally important. To explore the effects of nitric oxide (NO) and phosphorus (P) stress on the early symbiosis of truffles and host plants, different concentrations of exogenous NO donor sodium nitroprusside (SNP) and P were applied to *Carya illinoinensis* seedlings inoculated with the Chinese black truffle (*Tuber indicum*). The growth of *T. indicum*-mycorrhized seedlings and their mycorrhizal colonization rate were investigated. Additionally, the denitrifying bacterial community harboring NO reductase (*norB*) genes and the fungal community in the rhizosphere of the host were analyzed by high-throughput sequencing. The results showed that the colonization rate of *T. indicum* was significantly influenced by SNP treatments and P stress, with the highest level being obtained when the SNP was 100 μmol/L under low P stress (5 μmol/L). Treatment with 100 μmol/L SNP alone also increased the colonization rate of *T. indicum* and had positive effects on the plant height, stem circumference, biomass, root-shoot ratio and root POD activity of the seedlings at different times after inoculation. Under low P stress, the 100 μmol/L SNP increased the richness of the *norB*-type denitrifying bacterial community. Interestingly, the diversity and richness of *norB*-type denitrifying bacteria were significantly positively correlated with the colonization rate of *T. indicum*. SNP treatments under low P stress altered the abundance of some dominant taxa such as *Alphaproteobacteria*, *Gammaproteobacteria*, *Pseudomonas*, *Ensifer*, and *Sulfitobacter.* Evaluation of the fungal community in the rhizosphere revealed that 100 μmol/L SNP treatment alone had no noticeable effect on their richness and diversity, but it did shape the abundance of some fungi. *Buellia*, *Podospora, Phaeoisaria, Ascotaiwania*, and *Lophiostoma* were more abundant following exogenous NO application, while the abundance of *Acremonium, Monographella*, and *Penicillium* were decreased. Network analysis indicated that *T. indicum* was positively and negatively correlated with some fungal genera when treated with 100 μmol/L SNP. Overall, these results revealed how exogenous NO and P stress influence the symbiosis of truffles and host plants, and indicate that application of SNP treatments has the potential for ectomycorrhizal synthesis and truffle cultivation.

## Introduction

*Tuber* spp., commonly known as truffles, are ascomycete fungi that form ectomycorrhizae in a symbiotic relationship with plant roots and are prized for their hypogeous, edible fruiting bodies which adds a unique flavor to dishes (Kues and Martin, 2011; Vahdatzadeh et al., 2015). The interest in artificial cultivation of truffles has increased because of both the scarcity of truffle resources and reports of them being a source of polysaccharides with antitumor activity (Luo et al., 2011; Pereira et al., 2013; Zhao et al., 2014; Schmidberger and Schieberle, 2017). At present, synthesis of truffle-colonized seedlings and establishment of truffle plantations are the main methods of truffle cultivation (Deng et al., 2014). *Tuber indicum*, which is morphologically and phylogenetically similar to *T. melanosporum*, is the major commercial species of black truffle in China (Geng et al., 2009; Liu et al., 2011). *Carya illinoinensis* is an economically important nut tree native to North America, which is now cultivated worldwide (Benucci et al., 2012; Marozzi et al., 2017). Accordingly, utilizing *C. illinoinensis* as the host plant for *T. indicum* would have many practical and economic benefits and it has been shown that the ectomycorrhizae of *T. indicum* have now been successfully cultivated with *C. illinoinensis* (Bonito et al., 2011). Although the mycorrhization of these two organisms has been successfully accomplished, determining how to increase the ectomycorrhizal colonization levels and physiological and molecular mechanisms in response to this symbiont formation requires further exploration.

Ectomycorrhizae play an important role in P cycling of the rhizosphere (Cumming et al., 2015; Liu et al., 2018). Many studies have shown that ectomycorrhizae can contribute to the P absorption of plant roots, especially when P is scarce in the rhizosphere of the soil (Núñez et al., 2008; Bortier et al., 2018; Köhler et al., 2018). The content of P in the environment has been verified to impact the growth of ectomycorrhizal fungi and their colonization (Jones et al., 1990; Xue et al., 2008; Kluber et al., 2012). NO can affect the growth and development of plants as a signaling molecule that participates in many physiological processes, including seed germination, leaf growth, lateral root growth, stomatal movement and response to various biotic and abiotic stresses (Baudouin, 2011; Corpas and Barroso, 2015). Some reports have shown that NO can be produced in much higher levels in plants roots after colonization by arbuscular mycorrhizal fungi (Calcagno et al., 2012; Corpas and Barroso, 2015), which indicates that NO is important during the process of mycorrhizal colonization. In addition, evidence of the involvement of NO in plant responses to low P stress has been obtained (Simontacchi et al., 2015). As an exogenous NO donor, sodium nitroprusside (SNP) has commonly been used to explore the effects of NO on the physiology of many plants. However, it is still not known if SNP can affect the colonization levels of ectomycorrhizal fungi such as truffles on host plant root systems. Moreover, the effects of exogenous NO on the ectomycorrhizal synthesis of *T. indicum* and on the symbiotic system of *C. illinoinensis* with *T. indicum* under P stress conditions have not yet been reported.

In terrestrial ecosystems, ectomycorrhizal fungi including truffles have an important ecological function. A variety of microbial communities are involved in the lifecycle of truffles, and these play important roles in the truffle ectomycorrhizae and ascocarp formation, while also contributing to their aroma (Splivallo et al., 2015; Vahdatzadeh et al., 2015). Moreover, truffles have been predicted to influence soil microbial communities because of the formation of a brûlé (an area devoid of herbaceous cover) (Streiblova et al., 2012; Mello et al., 2013; Li et al., 2018). Our previous studies have also indicated that *T. indicum* shapes the bacterial and fungal communities in the ectomycorrhizosphere of *P. armandii* and *Q. aliena* (Li et al., 2017, 2018). Denitrification is a key nitrogen removal process that can produce NO, N_2_O, and N_2_, and different bacteria including bacteria harboring NO reductase (*norB*) genes can perform this process (Yunfu et al., 2017). Considering that NO may play an important role in the mycorrhizal colonization process of truffles and exogenous NO treatment was provided in this study, bacteria harboring *norB*-genes were selected for analysis, rather than 16S rRNA genes. Although many previous studies have investigated the microbial communities associated with truffles (Antony-Babu et al., 2014; Benucci and Bonito, 2016; Deveau et al., 2016; Fu et al., 2016), the specific roles of these microbial communities and the interaction between these microbes and truffle ectomycorrhizae is unclear, as are the conditions that occur under exogenous NO and P stress.

In this study, different concentrations of exogenous NO and P were provided to the *C. illinoinensis* seedlings inoculated and uninoculated with *T. indicum.* The colonization rate and the host plant growth and physiology were assessed. Additionally, high-throughput sequencing was used to analyze the *norB*-type denitrifying bacterial community of the rhizosphere soil. Next, to further explore the effects of NO alone on this symbiotic system, a suitable concentration of NO was selected for application to the colonized seedlings. The colonization rate, seedling growth and fungal communities of the rhizosphere soil were subsequently investigated from month 1 to 6 after inoculation. To our knowledge, this is the first study to explore the effects of exogenous NO and P stress on the ectomycorrhizal colonization of truffles and on the symbiotic system of host plants with truffles, with the goal of learning more about the physiological and molecular mechanism response to this symbiont formation under different conditions to improve the artificial cultivation of truffles.

## Materials and Methods

### *C. illinoinensis* Seedling Cultivation and *T. indicum* Inoculation

*Carya illinoinensis* seeds obtained from Yangbi County, China were first sterilized by soaking in 0.1% potassium permanganate solution for 2 h. Next, washed seeds were sown in sterilized nursery substrate composed of vermiculite, perlite, and water (volume ratio of 1:1:1) (Li et al., 2017). After 3 months, seedlings that were growing well were selected for transplantation into separate plastic containers filled with 1 L of sterilized cultivation substrate. There were two kinds of cultivation substrate prepared, Substrate I and Substrate II. Substrate I was composed of nutrient-poor sand, and Substrate II consisted of organic soil, vermiculite and water (volume ratio 1:1:0.5). The two cultivation substrates were autoclaved for 90 min at 121°C before use (Li et al., 2017, 2018). Truffle inoculation was performed when the seedlings were transplanted.

*Tuber indicum* was from Yanbian County, China. The truffle inoculum was prepared as previously described (Li et al., 2018). Briefly, 75% alcohol was used to disinfect the surface of the truffle ascocarps, after which they were pulverized and blended to spore powder. Next, 2 and 1 g of spore powder was inoculated into Substrate I and Substrate II, respectively, surrounding the roots of each *C. illinoinensis* seedling. There were 36 and 42 inoculated *C. illinoinensis* seedlings cultivated in Substrate I and Substrate II, respectively. Additionally, 3 uninoculated seedlings were cultivated in Substrate I. All seedlings were cultivated in a greenhouse under the same conditions with a clean environment and appropriate temperature and moisture content of the substrate.

### Experimental Design

#### Exogenous NO Treatment Combined With P Stress

Seedlings cultivated in Substrate I were subjected to exogenous NO treatment combined with P stress (Supplementary Table S1a).

After *T. indicum* inoculation, the exogenous NO donor SNP with four different concentrations (0, 10, 100, and 1000 μmol/L) was applied to the inoculated *C. illinoinensis* seedlings every 15 days, while uninoculated seedlings were treated with 0 μmol/L SNP. Samples were treated with 100 mL SNP per pot at each treatment time, half of which was applied to the cultivation substrate, while the remainder was sprayed on the leaf surface. The inoculated seedlings treated with 0, 10, 100, and 1000 μmol/L SNP were denoted S0, S1, S2, and S3, respectively.

When the seedlings were treated with different concentrations of SNP, they were also treated with different levels of P. Briefly, modified Hoagland nutrient solution containing three different concentrations of P was prepared (0, 5, and 2000 μmol/L). The P originated from KH_2_PO_4_ and the final concentration of each element in the nutrient solution except P is shown in Supplementary Table S2. The inoculated *C. illinoinensis* seedlings that were irrigated with 0, 5, and 2000 μmol/L P nutrient solution were denoted as the no P treatment (P_0_), low P treatment (P_5_), and high P treatment group (P_2000_), respectively. Uninoculated seedlings treated with 0 μmol/L SNP were only irrigated with 0 μmol/L P nutrient solution and were assigned to CK group. Overall, there are 12 treatments (excluding CK): P_0_S_0_, P_0_S_1_, P_0_S_2_, P_0_S_3_, P_5_S_0_, P_5_S_1_, P_5_S_2_, P_5_S_3_, P_2000_S_0_, P_2000_S_1_, P_2000_S_2_, and P_2000_S_3_. Each treatment contained at least three *C. illinoinensis* seedlings, all of which were timely irrigated with corresponding modified Hoagland nutrient solution, while sterile water was periodically applied to keep the cultivation substrate moist.

#### Exogenous NO Treatment Only

Exogenous treatment of only NO was applied to seedlings that were cultivated in Substrate II (Supplementary Table S1b). To further investigate the effects of only NO on the growth of *C. illinoinensis* seedlings with *T. indicum* colonization in the early symbiotic stage, the appropriate concentration (100 μmol/L) of SNP was applied alone as described above. Overall, half of the inoculated seedlings cultivated in Substrate II were treated with 100 mL of 100 μmol/L SNP every 15 days after inoculation of *T. indicum* until day 90 (SNP treatment), while the remaining inoculated seedlings were treated with an equal amount of water at the same time (Control-M treatment). The seedlings in these two treatments were irrigated with water every 2–3 days to keep the cultivation substrate moist.

#### Sampling Strategy and Analysis

After 4 months from inoculation, seedlings cultivated in Substrate I were observed, and samples were collected. The ectomycorrhizae of *C. illinoinensis* seedlings colonized by *T. indicum* were successfully detected by morphological analysis using a microscope. In each treatment, seedlings and their root system were harvested. Moreover, the rhizospheres soil of seedlings in CK and low P treatments (P_5_S_0_, P_5_S_1_, P_5_S_2_, and P_5_S_3_) were also collected aseptically. The mycorrhizal colonization rate was determined by counting the number of root segments colonized by *T. indicum* under a stereomicroscope based on the mycorrhizal fungal structures, with 30 root segments randomly selected in total for each seedling, which was finally expressed as: (root segments colonized by *T. indicum*/total observed root segments) × 100% (Andres-Alpuente et al., 2014). The plant morphology and physiology was determined immediately after the collection of seedlings and their roots. The rhizosphere soil samples were stored at −80°C prior to high-throughput sequencing of the *norB*-type denitrifying bacterial community.

For the seedlings of two treatments cultivated in Substrate II, their root systems and rhizosphere soil were collected every month after inoculation and used to determine the plant morphology and physiology, as well as for high-throughput sequencing of the fungal communities. Samples harvested at 0, 1, 2, 3, 4, 5, and 6 months after inoculation were denoted M0, M1, M2, M3, M4, M5, and M6, respectively. The ectomycorrhizae in samples from each month were detected and the colonization rate of *T. indicum* was calculated at month 6.

The whole experimental design and sampling strategy in this study can be seen in Supplementary Table S1. Three biological samples in each treatment were used for analysis, including the analysis of plant physiology, colonization rate and microbial communities.

### Determination of Plant Morphology and Physiology

The measured plant morphological and physiological indices included the plant height, stem circumference, root-shoot ratio, biomass, root activity, and superoxide dismutase (SOD) activity in roots and peroxidase (POD) activity in roots.

The plant height and stem circumference of *C. illinoinensis* seedlings were measured using a ruler and vernier caliper. Next, the seedlings were put in 100°C water for 20 min to halt respiration, then they were oven-dried at 75°C until constant weight for determination of the dry-weight, which was taken as the biomass. Next, the seedlings were divided into their underground and aboveground parts and the dry weights of the two parts were determined. The root-shoot ratio was expressed as the ratio of the dry weight of underground to aboveground parts of the seedlings (Maunoury-Danger et al., 2010).

The root activity was determined by the triphenyl tetrazolium chloride method as previously described (Zhang et al., 2012). The root SOD activity was determined by their ability to inhibit the photochemical reduction of nitro-blue tetrazolium (NBT) under light, and a unit of enzyme activity (U) was expressed as 50% inhibition of the NBT photoreduction (Fridovich, 2011). The determination of root POD activity was based on the theory that H_2_O_2_ can oxidize guaiacol under the catalysis of POD and form a tawny substance that can be detected using a spectrophotometer (Meloni et al., 2003). A unit of POD activity (U) was expressed as an absorbance change of 0.01 per minute at 470 nm.

### Soil DNA Extraction and PCR Amplification of *norB* Genes and ITS Genes

Total genomic DNA of the rhizosphere soil samples was extracted using a Power Soil^®^ DNA Isolation Kit (MoBio, Carlsbad, CA, United States) according to the manufacturer’s instructions. The extracted DNA was detected by 0.8% agarose gel electrophoresis and quantified by ultraviolet spectrophotometry.

For the DNA extracted from the soil samples of the P_5_S_0_, P_5_S_1_, P_5_S_2_, P_5_S_3_, and CK treatments (each treatment was performed in triplicate), the *norB* gene was amplified with the universal primers cnorB2F (5′-GACAAGNNNTACTGGTGGT-3′) and cnorB6R (5′-GAANCCCCANACNCCN GC-3′). The PCR reaction mix was 25 μL, which included the DNA template (2 μL), reaction buffer (5 μL), GC buffer (5 μL), 2 μL dNTPs (2.5 mmol L^–1)^, 1 μL forward primer (10 μmol L^–1^), 1 μL reverse primer (10 μmol L^–1^), 0.25 μL Q5 DNA polymerase, and 8.75 μL ddH_2_O. The cycling conditions were as follows: initial denaturation at 98°C for 2 min, followed by denaturation at 98°C for 15 s, and annealing at 55°C for 30 s, extension at 72°C for 30 s, after which samples were subjected to final extension at 72°C for 5 min. For the DNA extracted from the soil samples in Substrate II, the ITS1 region was amplified using primers ITS1 (5′-GGAAGTAAAAGTCGTA ACAAGG-3′) and ITS2 (5′-GCTGCGTTCTTCATCGATGC-3′). The PCR products were checked by 2% agarose gel electrophoresis and the target fragments were recovered using an Axygen Axy Prep DNA Gel Extraction kit (AxyGen Biosystems, United States). The recovered PCR products obtained from three technical replicates were combined in equidense ratios for each sample and purified with a Qiagen Gel Extraction Kit (Qiagen, Hilden, Germany). The PCR products were then quantified using a Quant-iT PicoGreen dsDNA Assay Kit (P7589, Invitrogen). An Illumina TruSeq Nano DNA LT Sample Prep Kit (Illumina, San Diego, CA, United States) was used to generate PCR amplicon libraries, after which the library quality was assessed with Agilent High Sensitivity DNA Kit (Agilent Technologies, Inc., United States).

### Illumina MiSeq High-Throughput Sequencing and Data Analysis

High throughput sequencing was conducted by Personal Biotechnology, Co., Ltd. (Shanghai, China) on an Illumina MiSeq sequencing platform. The overlapping paired-end reads were assembled using PEAR software and poor-quality sequences were removed using QIIME (v1.8.0) and USEARCH (v5.2.236) (Caporaso et al., 2010; Luo et al., 2017). High-quality sequences with 97% similarity were assigned to operational taxonomic units (OTUs) using UCLUST. The taxonomic information of *norB*-denitrifying bacteria was obtained using Ribosomal Database Project (RDP) (Release 11.1^1^) and the fungal sequences were taxonomically classed using UNITE database (Release 5.0^2^) (Wang et al., 2007; Edgar, 2010). The alpha and beta diversity of *norB*-type denitrifying bacterial and fungal communities were respectively analyzed using QIIME (v1.8.0). The alpha diversity of the species complexity of each sample was determined using the Chao1, ACE, Shannon, and Simpson indices. The beta diversity was determined by non-metric multidimensional scaling (NMDS) using R software, which reflects the differences in microbial communities among groups. Permutational multivariate analysis of variance (PERMANOVA) was performed by QIIME accompanied with NMDS. Linear discriminant analysis effect size analysis was used to respectively reveal the bacterial and fungal taxa at all taxonomic levels with significantly differential abundance between groups, which was carried out by Galaxy online analysis platform^3^. Network analysis for investigation of the interactions between the dominant genera was also performed using Mothur software (Schloss et al., 2009).

All of the raw sequencing data used in this study were submitted to the NCBI Sequence Read Archive (SRA) database with the accession number PRJNA544895/SRP199549.

### Statistical Analyses

Statistical analyses were performed using SPSS v22.0 (IBM, Inc., Armonk, NY, United States). The data were analyzed by one-way analyses of variance (ANOVAs) and independent *t-*tests, and the results reported were the means ± standard deviation (SD) of three biological replicates for each treatment. The least significant difference (LSD) test was performed using *P* < 0.05 as the threshold. Spearman’s correlation coefficient (rho) was calculated using SPSS 22.0.

## Results

### Effects of *T. indicum* Colonization on *C. illinoinensis* Seedlings

**FIGURE 1:**
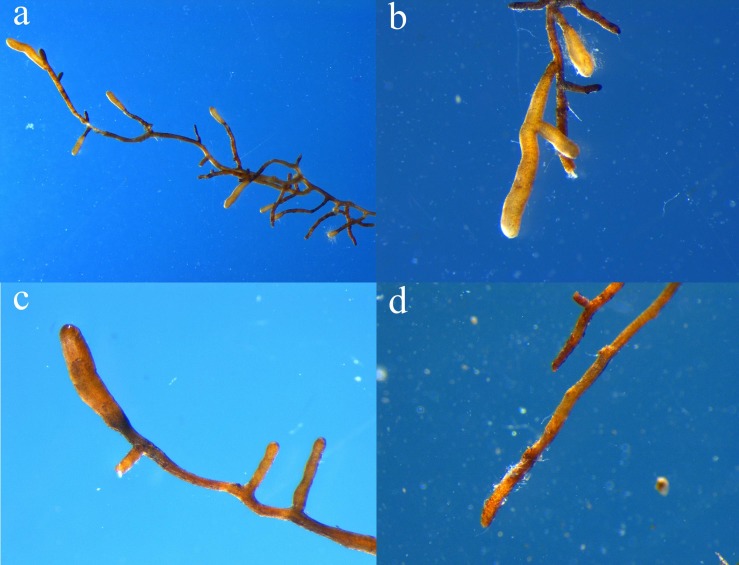
Ectomycorrhizae of *Carya illinoinensis* seedlings with *Tuber indicum* **(a–c)** and the roots of *C. illinoinensis* seedlings that were not colonized by *T. indicum* **(d)**.

Four months after inoculation, mycorrhization was successfully detected on the inoculated *C. illinoinensis* seedlings, while other uninoculated seedlings (CK) had not been colonized by truffles based on morphological evidence (Figure 1). Comparison of the P_0_S_0_ treatment and CK revealed that *T. indicum* inoculation significantly increased the plant height and POD activity in roots, but significantly decreased the root SOD activity (*P* < 0.05) (Table 1). The root activity and stem circumference were higher in the P_0_S_0_ treatment than in CK, but not significantly. *T. indicum* inoculation had no noticeable effect on the root-shoot ratio and biomass.

**TABLE 1:** The colonization rate of *T. indicum* and the growth of *C. illinoinensis* seedlings under different concentrations of exogenous NO donor SNP and P treatments.

**Treatments**	**Colonization rate**	**Root activity μg/(g⋅h) FW**	**SOD activity U/g FW**	**POD activity U/(g⋅min) FW**	**Root-shoot ratio**	**Biomass (g)**	**Plant height (mm)**	**Stem circumference (mm)**
CK	–	45.15 ± 2.95de	5.10 ± 0.11abc	148.16 ± 4.53e	1.91 ± 0.10a	3.56 ± 0.38abcd	294.67 ± 7.77e	4.19 ± 0.20bcd
P_0_S_0_	0.37 ± 0.02a	67.78 ± 4.68cd	4.26 ± 0.52d	462.66 ± 86.38a	1.65 ± 0.33ab	3.53 ± 0.56abcd	364.33 ± 35.72abcd	4.66 ± 0.83abcd
P_0_S_1_	0.42 ± 0.03b	61.49 ± 10.86cd	3.47 ± 0.51f	304.00 ± 42.56bc	1.23 ± 0.35b	2.54 ± 1.34cd	334 ± 60.70cde	4.39 ± 0.26abcd
P_0_S_2_	0.57 ± 0.03c	147.93 ± 39.85a	4.19 ± 0.28de	135.66 ± 58.7e	1.42 ± 0.16ab	3.06 ± 0.39abcd	351.67 ± 22.59bcde	4.12 ± 0.32cd
P_0_S_3_	–	120.47 ± 23.57b	3.62 ± 0.29ef	152.00 ± 42.56e	1.87 ± 0.15a	3.52 ± 0.79abcd	318 ± 28.16de	4.33 ± 0.20abcd
P_5_S_0_	0.44 ± 0.01b	68.26 ± 6.09cd	5.50 ± 0.54a	149.33 ± 24.58e	1.90 ± 0.28a	3.77 ± 0.56abc	386.33 ± 48.23abc	4.56 ± 0.06abcd
P_5_S_1_	0.56 ± 0.02c	74.71 ± 10.75c	5.51 ± 0.17cd	143.66 ± 38.17e	1.70 ± 0.44ab	3.83 ± 0.75abc	407 ± 17.44ab	4.76 ± 0.31ab
P_5_S_2_	0.81 ± 0.03d	82.81 ± 2.99e	5.39 ± 0.20a	356.66 ± 139.01b	1.44 ± 0.28ab	2.36 ± 0.14d	359.67 ± 17.01abcd	4.44 ± 0.20abcd
P_5_S_3_	–	28.89 ± 3.58cd	5.15 ± 0.27ab	242.66 ± 20.59cde	1.42 ± 0.27ab	3.13 ± 0.30abcd	412.67 ± 17.67a	4.72 ± 0.17abc
P_2000_S_0_	0.19 ± 0.03e	66.15 ± 12.2cd	5.30 ± 0.38ab	186.66 ± 22.03de	1.46 ± 0.57ab	2.89 ± 0.48bcd	412.67 ± 56.22a	4.08 ± 0.24d
P_2000_S_1_	0.23 ± 0.04f	69.78 ± 6.65cd	5.24 ± 0.34ab	210.33 ± 47.01cde	1.53 ± 0.56ab	4.32 ± 1.63a	310.33 ± 31.37de	4.89 ± 0.47a
P_2000_S_2_	0.63 ± 0.02g	67.47 ± 21.54cd	5.26 ± 0.20ab	290.00 ± 112.2bcd	1.86 ± 0.63a	2.76 ± 1.12cd	321.33 ± 26.73de	4.11 ± 0.58cd
P_2000_S_3_	–	62.04 ± 10.40cd	4.73 ± 0.46bcd	181.00 ± 49.15de	1.81 ± 0.02ab	4.13 ± 0.45ab	347 ± 39.51cde	4.31 ± 0.21abcd

*Each value is the mean of three replicates (± SD). Values followed by different lowercase letters indicate significant differences (*P* < 0.05) between samples in a row. NO, nitric oxide; SNP, exogenous NO donor sodium nitroprusside; P, phosphorus; FW, fresh weight. CK, uninoculated *C. illinoinensis* seedlings with 0 μmol/L SNP and P supply. P_0_, P_5_, and P_2000_ represent *C. illinoinensis* seedlings colonized by *T. indicum* treated with 0, 5, and 2000 μmol/L P, respectively. S_0_, S_1_, S_2_, and S_3_ represent *C. illinoinensis* seedlings colonized by *T. indicum* treated with 0, 10, 100, and 1000 μmol/L SNP, respectively.*

### Effects of Exogenous NO Combined With P Stress on *C. illinoinensis* Seedlings Colonized by *T. indicum*

The colonization rate of *T. indicum* and the physiological indices of *C. illinoinensis* seedlings were significantly affected by different concentrations of exogenous NO and P (Table 1). Under the same P concentration, the colonization rate of *T. indicum* on *C. illinoinensis* seedlings significantly increased as the SNP concentration increased from 0 to 100 μmol/L (*P* < 0.05), but when the SNP concentration increased to 1000 μmol/L, there were no ectomycorrhizae successfully detected. Under the same concentration of SNP (except 1000 μmol/L), the colonization rate was significantly higher when the P concentration was 5 μmol/L (*P* < 0.05) and reached a maximum of 81 ± 3% in P_5_S_2_ treatment.

The different concentrations of P had no noticeable effect on the plant height, stem circumference, root-shoot ratio or biomass of seedlings (Table 1). In the P_0_ and P_5_ treatments, no differences in these four indices under different SNP concentrations were observed, while in the P_2000_ treatments, the stem circumference and biomass were significantly higher when the SNP concentration was 10 μmol/L compared to when SNP was 0 and 100 μmol/L (*P* < 0.05).

The highest root activity was observed in the P_0_S_2_ treatment. Specifically, no significant differences were observed among the three different P levels when the SNP concentration was 0 or 10 μmol/L, but at 100 μmol/L, the root activity significantly decreased as the P concentration increased (*P* < 0.05). In the P_0_ and P_5_ treatments, root activity was significant higher when SNP was 100 μmol/L (*P* < 0.05), and there were no significant differences in P_2000_ treatments. The SOD activity of roots in the P_0_ treatments were significantly lower than in the P_5_ and P_2000_ treatments (*P* < 0.05). There was no noticeable effect of different SNP concentrations on root SOD activity. The maximum POD activity was observed in the P_0_S_0_ treatment, followed by the P_5_S_2_ treatment. In the P_5_ treatments, POD activity was significantly higher when the SNP was 100 μmol/L (*P* < 0.05) and there were no significant differences between P_2000_ treatments. Overall, the colonization rate and the physiological indices of the root system were higher when the SNP concentration was 100 μmol/L and the P content was low.

### Effects of Solely Exogenous NO (100 μmol/L SNP) on the Growth of *C. illinoinensis* Seedlings Colonized by *T. indicum*

**FIGURE 2:**
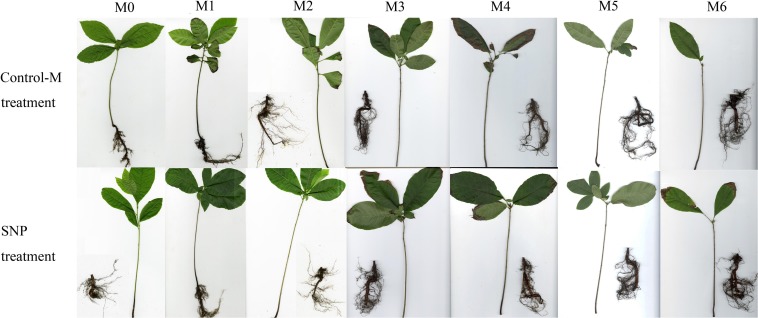
Morphology of inoculated *C. illinoinensis* seedlings and their root system with or without exogenous NO (100 μmol/L SNP). Control-M treatment, inoculated seedlings that did not receive the 100 μmol/L SNP treatment; SNP treatment, inoculated seedlings treated with 100 μmol/L SNP. M0, M1, M2, M3, M4, M5, and M6 represent seedlings harvested at month 0, 1, 2, 3, 4, 5, and 6, respectively, after *T. indicum* inoculation.

The morphology of the inoculated seedlings and their root systems are shown in Figure 2. The seedlings supplied by SNP grew better and had more lateral roots. The ectomycorrhizae of the two different treatments both occurred on the third month after inoculation, but the indicators of *T. indicum* colonization were more noticeable in the SNP treatment. On the sixth month after inoculation, the colonization rate of *T. indicum* reached 88 ± 2% on the seedlings to which 100 μmol/L SNP were applied, which was significantly higher than that of the seedlings in the Control-M treatment (62 ± 3%) (*P* < 0.05).

**FIGURE 3:**
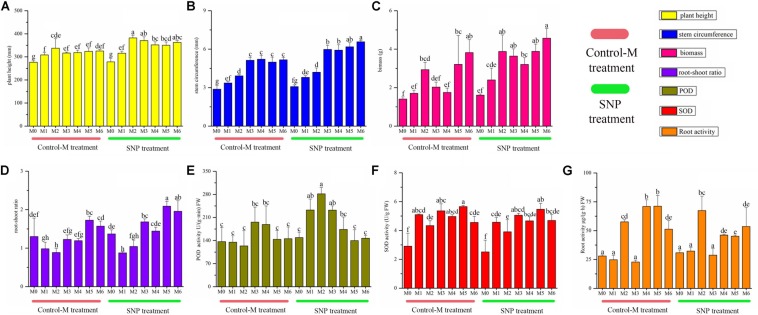
Growth indicators of inoculated *C. illinoinensis* seedlings with or without exogenous NO (100 μmol/L SNP) at different growth months after inoculation. **(A)** plant height, **(B)** stem circumference, **(C)** biomass, **(D)** root-shoot ratio, **(E)** POD activity in roots, **(F)** SOD activity in roots, **(G)** root activity. Each value is the mean of three replicates (±SD) in a treatment. Different lowercase letters indicate significant differences between all of the treatments in different months after inoculation (*P* < 0.05 in the LSD test of ANOVA). Control-M treatment: inoculated seedlings that did not receive 100 μmol/L SNP; SNP treatment: inoculated seedlings treated with 100 μmol/L SNP. M0, M1, M2, M3, M4, M5, and M6 represent seedlings harvested at month 0, 1, 2, 3, 4, 5, and 6, respectively, after *T. indicum* inoculation.

Sodium nitroprusside treatment significantly increased the plant height from the second month to the sixth month compared with the Control-M (*P* < 0.05), and the stem circumference became significantly thicker in the SNP treatment from the third to the sixth month (*P* < 0.05) (Figure 3). The biomass was higher in the SNP treatment, and significant differences were observed between the treatments on the third and fourth month (*P* < 0.05). SNP treatment also significantly increased the root-shoot ratio on the third, fifth and sixth month (*P* < 0.05). The POD activity in roots differed significantly between the two treatments on the first and second month, and was also higher in response to SNP treatment (*P* < 0.05). However, SNP treatment had no noticeable effect on the SOD activity in roots because there were no significant differences between the two treatments after inoculation. Root activity was significantly lower in response to SNP treatment during the fourth and fifth month (*P* < 0.05).

### Analyses of *norB*-Type Denitrifying Bacterial Communities

#### Alpha Diversity of *norB*-Type Denitrifying Bacteria in Rhizosphere Soil

**FIGURE 4:**
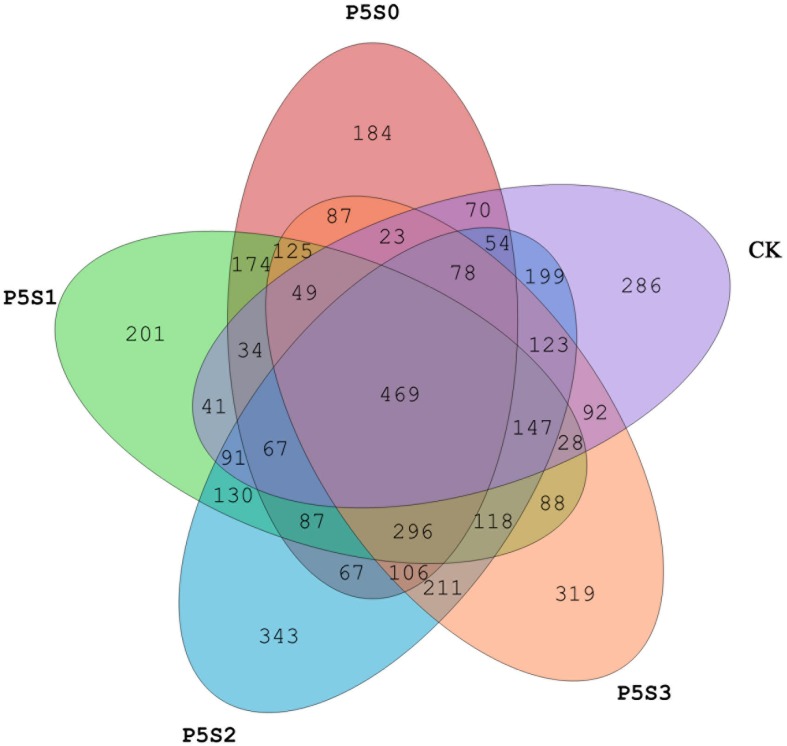
Shared and unique *norB*-type denitrifying bacterial operational taxonomic units (OTUs) among rhizosphere soil samples in CK treatment and in different SNP treatments under low P stress. CK, the rhizosphere soil of uninoculated *C. illinoinensis* seedlings that received 0 μmol/L SNP and P application. P_5_S_0_, P_5_S_1_, P_5_S_2_, and P_5_S_3_ represent the rhizosphere soil of *C. illinoinensis* seedlings colonized by *T. indicum* treated with 0, 10, 100, and 1000 μmol/L SNP, respectively, under low P stress (5 μmol/L).

Sequencing of the rhizosphere soils of the CK treatment and those treated with different concentrations of SNP under low P stress were yielded 947,615 high-quality sequences from all 15 samples after quality control, which were clustered into 4,387 OTUs (Supplementary Figure S1a). The Venn diagram revealed 469 shared OTUs among the samples of the five different treatments (Figure 4), with the number of unique OTUs in treatment P_5_S_2_ being highest, followed by that in the P_5_S_3_ treatment. The unique number of OTUs in P_5_S_0_ and P_5_S_1_ was even lower than that in CK.

The two diversity indices (Shannon and Simpson) showed no significant differences among the five treatments (Table 2), indicating that the effects of treatment with different concentrations of SNP on the diversity of *norB*-type denitrifying bacteria were not significant. The estimated richness indices (Chao1 and ACE) revealed that the *norB*-type denitrifying bacterial community richness was highest in the P_5_S_2_ treatment, and was significantly higher than that in the P_5_S_0_ and CK treatments (*P* < 0.05).

**TABLE 2:** The richness and diversity indices of *norB*-type denitrifying bacteria in rhizosphere soil of *C. illinoinensis* seedlings with different concentrations of exogenous NO donor SNP treatment under low P stress.

**Treatments**	**Simpson**	**Shannon**	**Chao1**	**ACE**
CK	0.99 ± 0.00a	7.76 ± 0.21a	1106.46 ± 224.79a	1115.67 ± 232.67a
P_5_S_0_	0.90 ± 0.13a	6.42 ± 1.52a	1142.76 ± 94.78a	1141.64 ± 110.57a
P_5_S_1_	0.94 ± 0.07a	6.87 ± 1.19a	1330.28 ± 173.28ab	1334.32 ± 168.06ab
P_5_S_2_	0.98 ± 0.00a	8.06 ± 0.03a	1641.35 ± 55.19b	1666.54 ± 66.02b
P_5_S_3_	0.93 ± 0.09a	6.93 ± 1.14a	1325.11 ± 359.71ab	1366.19 ± 362.22ab

*Each value is the mean of three replicates (± SD). Values followed by different lowercase letters indicate significant differences (*P* < 0.05) between samples in a row. NO, nitric oxide; SNP, exogenous NO donor sodium nitroprusside; P, phosphorus. CK, rhizosphere soil of uninoculated *C. illinoinensis* seedlings with 0 μmol/L SNP and P application. P_5_S_0_, P_5_S_1_, P_5_S_2_, and P_5_S_3_ represent the rhizosphere soil of *C. illinoinensis* seedlings colonized by *T. indicum* that were treated with 0, 10, 100, and 1000 μmol/L SNP, respectively, under low P stress (5 μmol/L).*

#### Taxonomic Composition of *norB*-Type Denitrifying Bacterial Communities

In the 15 samples from the five different treatments, a total of 10 phyla, 16 classes, 35 orders, 56 families, and 95 genera of *norB*-type denitrifying bacterial communities were detected. At the phylum level, *Proteobacteria* was the most abundant phylum, accounting for 98.33% (Supplementary Figure S2), followed by *Actinobacteria* (1.07%) and *Acidobacteria* (0.42%). The relative abundance of these three phyla showed no significant differences among the five treatments.

**FIGURE 5:**
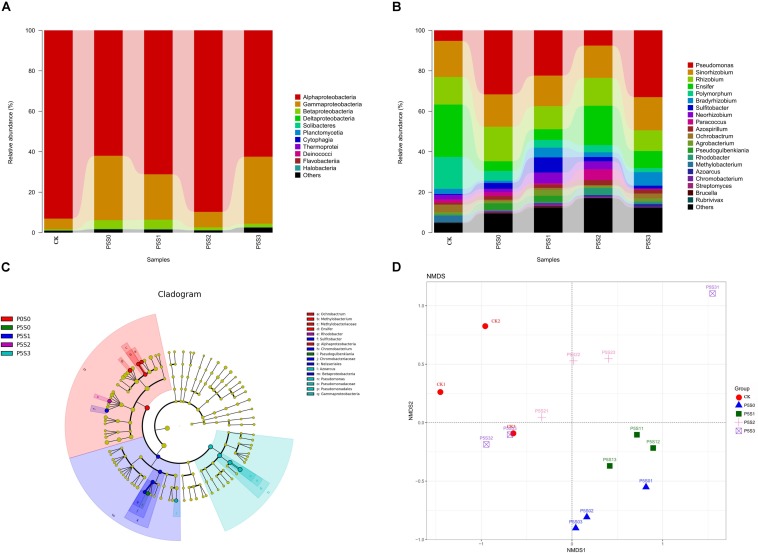
Taxonomic composition of *norB*-type denitrifying bacterial communities at the **(A)** class and **(B)** genus levels in rhizosphere soil of *C. illinoinensis* seedlings in CK treatment and in different SNP treatments under low P stress. **(C)** Cladogram based on linear discriminant analysis effect size (LEfSe) analysis (*P* < 0.05, LDA score > 2) showing significant differences in abundance of *norB*-type denitrifying bacterial taxa in different groups; **(D)** non-metric multidimensional scaling (NMDS) analysis of *norB*-type denitrifying bacterial communities in rhizosphere soil of *C. illinoinensis* seedlings with different treatments. All treatments were had three replicates. CK, rhizosphere soil of uninoculated *C. illinoinensis* seedlings that received 0 μmol/L SNP and P application. P_5_S_0_, P_5_S_1_, P_5_S_2_, and P_5_S_3_ represent the rhizosphere soil of *C. illinoinensis* seedlings colonized by *T. indicum* and treated with 0, 10, 100, and 1000 μmol/L SNP, respectively, under low P stress (5 μmol/L).

At the class level (Figure 5A), *Alphaproteobacteria* (75.70%), *Gammaproteobacteria* (20.07%), and *Betaproteobacteria* (2.50%) were the dominant taxa. Under low P stress, the abundance of *Alphaproteobacteria* gradually increased as the SNP concentrations increased from 0 to 100 μmol/L and then decreased when the SNP concentration was 1000 μmol/L. *Alphaproteobacteria* was significantly more abundant in the CK and P_5_S_2_ groups than in the P_5_S_0_ and P_5_S_3_ groups (*P* < 0.05). The abundance of *Gammaproteobacteria* was lowest in the CK and P_5_S_2_ groups, while the P_5_S_3_ treatment contained significantly more *Gammaproteobacteria* than the CK (*P* < 0.05). *Betaproteobacteria* was more abundant in the P_5_S_1_ group.

At the genus level (Figure 5B), the most abundant genera were *Pseudomonas* (19.97%), *Sinorhizobium* (16.27%), *Rhizobium* (13.20%), *Ensifer* (12.80%), *Rhodobacteraceae_unidentified* (8.70%), *Polymorphum* (5.96%), *Bradyrhizobium* (3.52%), and *Sulfitobacter* (2.88%). Under low P stress, *Pseudomonas* abundance gradually decreased as the SNP concentrations increased from 0 to 100 μmol/L, then increased to the maximum when the SNP concentration was 1000 μmol/L. The abundance of *Pseudomonas* in the P_5_S_3_ group was significantly higher than in the CK (*P* < 0.05) (Figure 5C). *Sinorhizobium* and *Rhizobium* showed no significant differences among the five treatments. *Ensifer* was significantly more abundant in the CK (*P* < 0.05) (Figure 5C), and the change in its abundance was contrary to that of *Pseudomonas. Polymorphum* was also significantly more abundant in CK (*P* < 0.05), while *Sulfitobacter* was significantly more abundant in the P_5_S_1_ group (*P* < 0.05) than in the other groups (Figure 5C).

#### Structural Differentiation and Network Associations of *norB*-Type Denitrifying Bacterial Communities

The differences in the *norB*-type denitrifying bacterial community structure among the five treatments were visualized by NMDS analysis (PERMANOVA, *P* = 0.001) (Figure 5D). The *norB*-type denitrifying bacterial community structures of the P_5_S_0_ and P_5_S_1_ treatment were similar and differed significantly from those of the other treatments. The community structure of the P_5_S_2_ treatment also differed from that of other treatments.

Among the top 50 genera of *norB*-type denitrifying bacterial communities, 36 showed correlations with others (Supplementary Figure S4a). *Pseudomonas* was negatively correlated with *Ensifer* and *Paracoccus*. *Sulfitobacter* was positively correlated with *Pseudogulbenkiania, Chromobacterium*, and *Anaeromyxobacter*, while it was negatively correlated with *Methylobacterium*.

#### Correlation Analysis Between *norB*-Type Denitrifying Bacterial Community and Colonization Rate of *T. indicum*

There were significant correlations between the colonization rate of *T. indicum* and the richness and diversity of *norB*-type denitrifying bacterial communities (*P* < 0.05) (Supplementary Table S3). Based on the Chao1 and ACE indices, colonization rate was positively correlated with the richness of the *norB*-type denitrifying bacterial communities. Additionally, the Shannon and Simpson indices indicated that colonization rate was positively correlated with the diversity of *norB*-type denitrifying bacterial communities.

### Analyses of Fungal Communities

#### Fungal Alpha Diversity in Rhizosphere Soil

**FIGURE 6:**
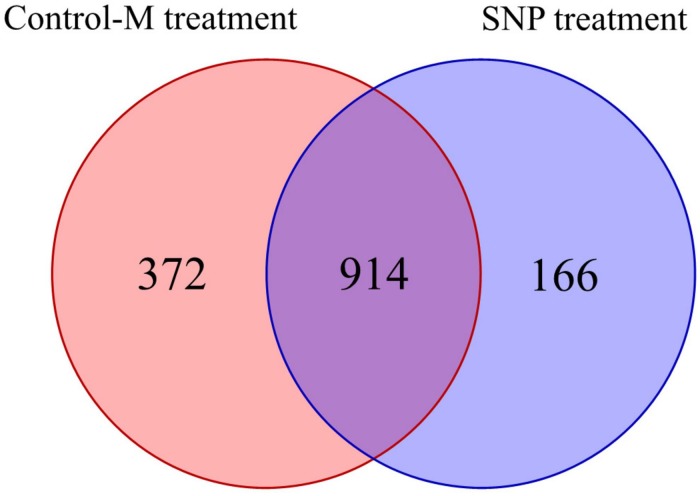
Shared and unique fungal operational taxonomic units among rhizosphere soil samples of inoculated *C. illinoinensis* seedlings with or without exogenous NO (100 μmol/L SNP) application. Control-M treatment, inoculated seedlings that did not receive 100 μmol/L SNP; SNP treatment, inoculated seedlings that received 100 μmol/L SNP.

Overall, 1,824,064 high-quality sequences were obtained from the 42 samples collected during different months after quality control procedures. These sequences were clustered into 1,452 OTUs in all, and the rarefaction curves of the fungal OTUs in different samples are shown in Supplementary Figure S1b. The Venn diagram displays the degree of overlap of the fungal OTUs between the samples in the two treatments (Figure 6). The number of the unique OTUs in the SNP treatment was 166, which was twofold lower than that in the Control-M treatment.

Based on the Chao1 and ACE indices, fungal community richness of the rhizosphere soil did not differ significantly between the SNP treatments and Control-M treatments during each month (Table 3). Additionally, the Simpson index indicated that fungal diversity did not differ significantly between the two different treatments during each month. The Shannon index indicated that the fungal diversity was lowest in the fourth month in the two different treatments, but was significantly higher in the Control-M treatment in the third month compared with the SNP treatment (*P* < 0.05). In general, the SNP treatment did not have any noticeable effect on the diversity and richness of fungal communities in rhizosphere soil at different growth times.

**TABLE 3:** The richness and diversity indices of fungal communities in rhizosphere soil of inoculated *C. illinoinensis* seedlings with or without exogenous NO (100 μmol/L SNP) application during different growth months.

**Treatments**		**Simpson**	**Shannon**	**Chao1**	**ACE**
M0	Control-M	0.91 ± 0.05a	4.95 ± 0.39ab	266.20 ± 26.72bc	264.06 ± 24.70a
	SNP	0.92 ± 0.02a	5.11 ± 0.63ab	301.52 ± 80.34abc	284.47 ± 53.80abc
M1	Control-M	0.90 ± 0.06ab	4.69 ± 0.26abc	262.35 ± 11.56bc	262.71 ± 10.93a
	SNP	0.87 ± 0.11ab	4.78 ± 0.81abc	299.83 ± 50.00abc	302.59 ± 52.85abc
M2	Control-M	0.93 ± 0.02a	5.10 ± 0.48ab	382.64 ± 28.95a	388.59 ± 26.33c
	SNP	0.84 ± 0.11ab	4.20 ± 0.91abcd	303.92 ± 45.85abc	310.10 ± 50.84abc
M3	Control-M	0.90 ± 0.07a	5.18 ± 1.56a	321.62 ± 152.39abc	320.60 ± 142.49abc
	SNP	0.85 ± 0.03ab	3.79 ± 0.48bcde	263.76 ± 78.18bc	267.17 ± 81.44a
M4	Control-M	0.64 ± 0.28c	3.07 ± 1.44de	254.77 ± 70.15bc	262.49 ± 70.52a
	SNP	0.64 ± 0.06c	2.76 ± 0.44e	244.25 ± 105.38c	243.88 ± 96.14a
M5	Control-M	0.85 ± 0.01ab	4.05 ± 0.15abcde	287.90 ± 43.41abc	288.71 ± 44.29abc
	SNP	0.88 ± 0.04ab	4.58 ± 0.33abc	369.21 ± 19.64ab	379.63 ± 24.03bc
M6	Control-M	0.78 ± 0.15abc	3.85 ± 1.00abcde	262.81 ± 43.27bc	271.03 ± 49.00ab
	SNP	0.72 ± 0.13bc	3.56 ± 0.75cde	307.52 ± 66.21abc	318.07 ± 67.84abc

*Each value is the mean of three replicates (± SD). Values followed by different lowercase letters indicate significant differences (*P* < 0.05) between samples in a row. NO, nitric oxide; SNP, exogenous NO donor sodium nitroprusside. Control-M treatment, the rhizosphere soil of inoculated *C. illinoinensis* seedlings that had no SNP application; SNP treatment, the rhizosphere soil of inoculated *C. illinoinensis* seedlings treated with 100 μmol/L SNP. M0, M1, M2, M3, M4, M5, and M6 represent rhizosphere soil harvested on month *0, 1, 2, 3, 4, 5*, and *6*, respectively, after *T. indicum* inoculation.*

#### Taxonomic Composition of Fungal Communities

**FIGURE 7:**
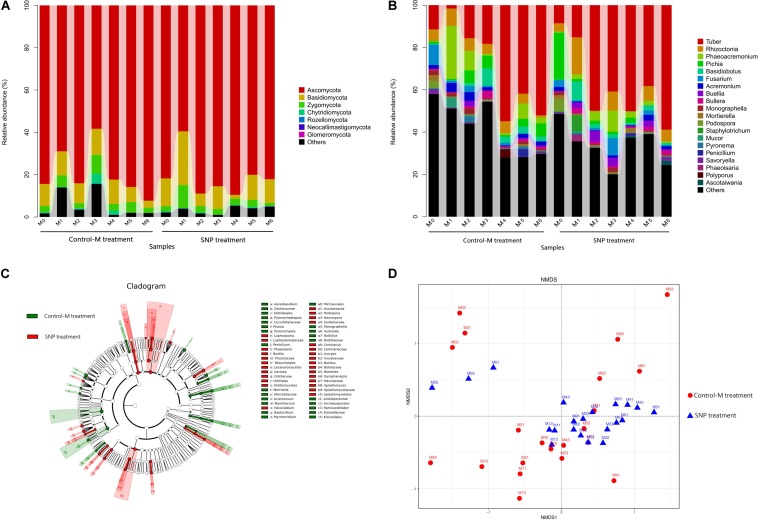
Taxonomic composition of fungal communities at the **(A)** phylum and **(B)** genus levels in rhizosphere soil of inoculated *C. illinoinensis* seedlings with or without 100 μmol/L SNP during different growth months. **(C)** Cladogram based on linear discriminant analysis effect size (LEfSe) analysis (*P* < 0.05, LDA score > 2) showing the significantly differentially abundant fungal taxa in the rhizosphere soil of inoculated *C. illinoinensis* seedlings with or without SNP application. **(D)** Non-metric multidimensional scaling analysis of fungal communities in rhizosphere soil of inoculated *C. illinoinensis* seedlings with or without SNP application during different growth months. All of the treatments were conducted with three replicates. Control-M treatment, inoculated seedlings that did not receive 100 μmol/L SNP application; SNP treatment, inoculated seedlings that received 100 μmol/L SNP. M0, M1, M2, M3, M4, M5, and M6 represent seedlings harvested at month 0, 1, 2, 3, 4, 5, and 6, respectively, after *T. indicum* inoculation.

Among the 42 samples of SNP and Control-M treatments, a total of 9 phyla, 30 classes, 84 orders, 177 families, and 291 genera were detected. At the phylum level, *Ascomycota* (80.26%) was the dominant fungal phylum, followed by *Basidiomycota* (10.41%) and *Zygomycota* (4.08%) (Figure 7A). The relative abundance of these three phyla showed no significant differences between the SNP treatments and Control-M treatments in each month.

At the class level, *Pezizomycetes* (41.06%), *Sordariomycetes* (18.32%), *Agaricomycetes* (10.53%), and *Saccharomycetes* (8.58%) were the dominant taxa (Supplementary Figure S3). The relative abundance of *Pezizomycetes* did not differ between the SNP treatments and Control-M treatments during each month. However, the SNP treatment significantly decreased the abundance of *Sordariomycetes* on the first month and decreased that of *Saccharomycetes* on the second month when compared with the Control-M treatment (*P* < 0.05).

At the genus level, the top 10 of the most abundant genera were *Tuber* (32.68%), *Rhizoctonia* (5.64%), *Phaeoacremonium* (4.93%), *Pichia* (3.81%), *Basidiobolus* (2.53%), *Fusarium* (2.27%), *Acremonium* (1.71%), *Buellia* (1.44%), *Bullera* (1.30%), and *Monographella* (0.98%) (Figure 7B). *Tuber* abundance was higher in the SNP treatment, but this difference was not significant. The relative abundance of *Tuber*, *Rhizoctonia*, *Phaeoacremonium*, and *Basidiobolus* did not differ significantly between the two treatments during each month. However, *Pichia* abundance was significantly lower in the SNP group than in the Control-M on the second month (*P* < 0.05). The abundance of *Fusarium* was also significantly lower in the SNP treatment on the first and second month (*P* < 0.05). In the SNP groups, *Tuber* showed significantly greater abundance from the second to the sixth month compared with month 0 and 1 (*P* < 0.05). However, in Control-M treatment, *Tuber* abundance increased from the fourth month, and was significantly more abundant compared with months 0, 1, 2, and 3 (*P* < 0.05).

#### Differentially Abundant Taxa and Network Associations of Fungal Communities

Linear discriminant analysis effect size (LEfSe) analysis was used to reveal the fungal taxa that showed significantly different abundance between the SNP and Control-M treatments (*P* < 0.05) (Figure 7C). At the phylum level, there were no differentially abundant phyla between treatments. At the class level, the samples of the SNP treatments contained significantly more *Orbiliomycetes* and *Lecanoromycetes.* At the family level, the relative abundances of *Physciaceae, Orbiliaceae, Boletaceae, Lophiostomataceae, Inocybaceae*, and *Cortinariaceae* were significantly higher in the SNP treatments, while the abundances of *Dothioraceae* and *Kickxellaceae* were significantly higher in the Control-M treatments. At the genus level, among the top 50 genera, *Buellia*, *Podospora*, *Phaeoisaria*, *Ascotaiwania*, and *Lophiostoma* were significantly more abundant in the SNP treatments while *Acremonium, Monographella*, and *Penicillium* were significantly more abundant in the Control-M treatments.

Among the top 50 genera, 39 showed correlations with others (Supplementary Figure S4b). *Tuber* was negatively correlated with *Archaeorhizomyces*, *Podospora* and *Penicillium*, but positively correlated with *Tricholoma.*

#### Structural Differentiation of Fungal Communities

Differences in fungal community structure among samples were visualized by NMDS analysis (PERMANOVA, *P* = 0.008) (Figure 7D). In SNP treatments, the fungal community structure of samples at month 0 differed obviously from those of other months. Analogously, in Control-M treatments, the fungal community structure of the samples at month 0, from month 0 to month 5, and at month 6 differed from each other. Comparison of the SNP treatments and Control-M treatments revealed that the fungal community structures of the two treatments differed in the same month, indicating that exogenous NO can shape the fungal community structure to a certain degree.

## Discussion

As ectomycorrhizal fungi, the successful and efficient synthesis of ectomycorrhizae is the basis for the artificial cultivation of truffles (Li et al., 2017). The ability of truffles to colonize plant roots and successfully form ectomycorrhizae can be affected by various abiotic and biotic factors such as soil properties, soil fertility, soil microorganisms, and vegetation (Slankis, 1974). Thus, the surrounding environment and management measures are important to the symbiosis of truffles and host plants. In this study, different concentrations of exogenous NO donor SNP and P were provided to the *C. illinoinensis* seedlings. The shifts in the colonization levels of *T. indicum*, in the growth of host plants, and in the associated microbes of rhizosphere soil were then investigated during the early symbiotic stage.

The colonization rate of truffles reflects the degree of mycorrhization (García-Montero et al., 2008). In our study, different concentrations of exogenous NO and P had significant effects on the colonization rate of *T. indicum* with *C. illinoinensis* seedlings. The colonization rate reached a high level (81 ± 3%) when SNP was 100 μmol/L under low P stress (5 μmol/L). Previous research showed that plants could be less dependent on ectomycorrhizae for P absorption when more soil P is available, and that ectomycorrhizal colonization may be greater under P-limited conditions (Kluber et al., 2012). When compared with the high P treatments (2000 μmol/L) in the present study, the colonization levels of *T. indicum* significantly increased under low P stress. However, excessive P deficiency (no P treatments) did not contribute to colonization of *T. indicum.* The trend in colonization levels was P_5_ > P_0_ > P_2000_. These findings indicated that the plants could adjust their root architecture in response to low P conditions (Niu et al., 2013). The growth of primary roots was inhibited and the development of lateral roots, cluster roots and root hair was promoted to improve the P uptake. This adjustment seemed to provide more attached sites for ectomycorrhizal fungi, which was beneficial to the colonization of truffles. NO in plants was demonstrated to participate in the response to low P conditions (Niu et al., 2013; Simontacchi et al., 2015). P deficiency enhanced NO accumulation in primary and lateral roots. Previous studies confirmed that the appropriate concentration of SNP could promote cluster roots proliferation and lateral root development (Lira-Ruan et al., 2013; Corpas and Barroso, 2015; Sun et al., 2015). In the present study, 100 μmol/L SNP was found to be optimal for *T. indicum* colonization under different P concentrations, and high concentrations of SNP could completely inhibit *T. indicum* colonization. Treatment of *C. illinoinensis* seedlings with 100 μmol/L SNP alone also significantly increased the colonization rate of *T. indicum* (88 ± 2%). In previous studies, the colonization rate of truffles was between approximately 40 and 60%, depending on the truffle species species and host plant (García-Montero et al., 2008; Geng et al., 2009; Benucci et al., 2012; Li et al., 2018). Therefore, this increase in colonization rate caused by 100 μmol/L SNP could be applied to the ectomycorrhizal synthesis of truffles and material exchange between mycorrhizal fungi and host plants, which may be useful in the artificial cultivation of truffles.

In addition to the colonization rate, the quality of the host plant also contributes to the success or failure of truffle crops (Andres-Alpuente et al., 2014). If plant growth was improved while the ectomycorrhizal level was not affected, the truffle yields may be better or earlier (Bonet et al., 2006). *T. indicum* inoculation significantly increased the plant height and root POD activity of *C. illinoinensis* seedlings in this study, but had negative effect on the root SOD activity. Previous research showed that the growth of *Pinus halepensis* seedlings could be improved by *T. melanosporum* inoculation and that the nutrient uptake of the seedlings was also improved (Dominguez et al., 2012). *T. indicum* colonization on several Chinese indigenous trees could also lead to better growth of the host, showing higher ground diameter increases, plant height, and biomass compared with the uninoculated seedlings (Hu, 2004), which was similar with our results. However, further analysis is needed to explain the decrease in root SOD activity. Under low P stress, the maximum root and POD activity was obtained when the SNP concentration was 100 μmol/L, which was consistent with the colonization rate. Evaluation of various abiotic stresses revealed that SNP with appropriate concentration could enhance the activity of the antioxidant system in plants, such as SOD and POD (Yang et al., 2012; Arora and Bhatla, 2015). The increase in POD activity indicated that 100 μmol/L SNP improved the ability of the host plants to cope with stress. However, under low P stress, SOD activity was highest at 10 μmol/L SNP, and improvement of SOD activity in response to exogenous NO was not as great as the improvement of POD activity. Many studies have shown that exogenous NO application promoted plant growth under various stresses; however, the effects of exogenous NO on plants that formed symbiotic relationships with ectomycorrhizal fungi have rarely been reported (Dong et al., 2014; Liu et al., 2014; Kaya and Ashraf, 2015). In the present study, application of only 100 μmol/L SNP to inoculated *C. illinoinensis* seedlings induced positive effects on plant height, stem circumference, biomass, root-shoot ratio, and POD activity of seedlings, but the variations in these indicators were not synchronous during the 7 months after inoculation. Therefore, treatment with 100 μmol/L SNP could improve the growth of host plants colonized by truffles to a certain degree; however, the effect of SNP on the artificial cultivation of truffles and fructification requires further verification in the field.

Rhizosphere soil microbes play important roles in ecological environments associated with truffles, contributing to ectomycorrhizae synthesis and truffle production, as well as the formation of truffle aroma (Splivallo et al., 2015; Vahdatzadeh et al., 2015). Moreover, microbes in rhizosphere soil participate in plant growth as well as the plant tolerance to disease and abiotic stress (Choudhary, 2012). Using high-throughput sequencing, the effects of exogenous NO at different concentrations under low P stress on bacteria harboring *norB*-type genes in rhizosphere soil were analyzed in this study. Some studies have reported that NO was frequently involved in the early basal signaling of interactions between plant roots and bacteria, which greatly influenced the root growth patterns and the accumulation of major nutrients (Simontacchi et al., 2015; Vaishnav et al., 2018). NO was also found to promote the formation of biofilms in bacteria (Vaishnav et al., 2016). Under low P stress, the exogenous NO did not influence the diversity of *norB*-type denitrifying bacteria in the present study, but did increase their richness when 100 μmol/L SNP was applied. Interestingly, the diversity and richness of *norB*-type denitrifying bacteria were significantly correlated with the colonization rate of *T. indicum.* This indicated that an interactive network may exist among the NO, *norB*-type denitrifying bacteria community and the colonization of truffles. Many studies have investigated the role of NO in symbiotic interactions, and exogenous NO has been reported to promote the establishment of the PGPR, i.e., *Pseudomonas simiae*, strain, which contributed to better colonization and plant growth under saline conditions (Vaishnav et al., 2016). However, the role of NO in symbiotic interactions of ectomycorrhizal fungi is not clear. In the present study, exogenous NO affected some dominant populations of *norB*-type denitrifying bacteria under low P stress. *Alphaproteobacteria* was more abundant while *Gammaproteobacteria* was less abundant when SNP was applied at 100 μmol/L. *Alphaproteobacteria* and *Gammaproteobacteria* comprised the predominant components of the bacterial communities of truffles (Barbieri et al., 2007; Li et al., 2017). The abundance of the *Pseudomonas* genus was P_5_S_3_ > P_5_S_0_ > P_5_S_1_ > P_5_S_2_, which was contrary to the colonization rate. However, *Pseudomonas* was reported to play a role in ectomycorrhizal symbiosis and *P. fluorescens* is believed to be important to growth and truffle mycorrhizal synthesis (Dominguez et al., 2012; Li et al., 2017). However, further study is needed to explain these phenomena and the interactions of exogenous NO and the denitrifying bacteria associated with truffles.

To date, the effects of exogenous NO on fungal communities in rhizosphere soil have rarely been reported to date. In this study, the effects of only exogenous NO (100 μmol/L SNP) on soil rhizosphere fungi associated with truffles were investigated. Not surprisingly, *Tuber* was the dominate genus, accounting for 32.36%. These results indicated that exogenous NO did not significantly influence the abundance of *T. indicum* mycelia during the first 7 months after inoculation. However, the significant increase in *Tuber* abundance occurred earlier in exogenous NO treatments, which seems to be beneficial to truffle colonization. Previous studies showed that truffle inoculation reduced fungal richness and diversity in the roots and surrounding soil (Li et al., 2017, 2018). No significant effects of exogenous NO on the fungal richness and diversity in rhizosphere soil were observed in this study. NO has been shown to protect roots against further aggression from phytopathogens (Compant et al., 2010). In addition, *Buellia*, *Podospora, Phaeoisaria, Ascotaiwania*, and *Lophiostoma* were found to be more abundant because of exogenous NO application, while the abundance of *Acremonium, Monographella*, and *Penicillium* decreased. Network analysis provides an understanding of the potential interactions in microbial communities, and may identify keystone populations (Gu et al., 2018). During the early symbiotic stage, *Tuber* was positively correlated with *Tricholoma*, but negatively correlated with *Archaeorhizomyces*, *Podospora*, and *Penicillium* when 100 μmol/L SNP was provided. These fungal communities may be closely related to the growth of truffles under NO application.

## Conclusion

Both exogenous NO and P stress affected the ectomycorrhizal synthesis of *T. indicum* and the growth of host seedlings, with the shift of colonization rate, plant physiology, and some microbial communities in the rhizosphere, which could have potential application in the artificial cultivation of truffles in the future. Also, the mechanism of how exogenous NO and P stress affect the symbionts of truffles and the host also needs to be further explored.

## Data Availability Statement

The datasets generated for this study can be found in the NCBI Sequence Read Archive (SRA) database with the accession number PRJNA544895/SRP199549.

## Author Contributions

XL, XZ (first author), and XZ (last author) conceived and designed the experiments. CW, ZK, and LY performed the experiments. XZ (first author) and XL wrote and revised the manuscript. All of the authors approved the final version of the manuscript.

## Conflict of Interest

The authors declare that the research was conducted in the absence of any commercial or financial relationships that could be construed as a potential conflict of interest.

## Footnotes

1 http://rdp.cme.msu.edu/

2 https://unite.ut.ee/

3 http://huttenhower.sph.harvard.edu/galaxy/
