# Supplementary material for: Exogenous Nitric Oxide and Phosphorus Stress Affect the Mycorrhization, Plant Growth, and Associated Microbes of Carya illinoinensis Seedlings Colonized by Tuber indicum
Source: Front Microbiol. 2019 Nov 13;10:2634. doi: 10.3389/fmicb.2019.02634 (PMC6863891; doi:10.3389/fmicb.2019.02634)
Supplement: Supplementary file 1 [file Data_Sheet_1.pdf]

Supplementary Material

1 Supplementary Figures and Tables

1.1 Supplementary Figures

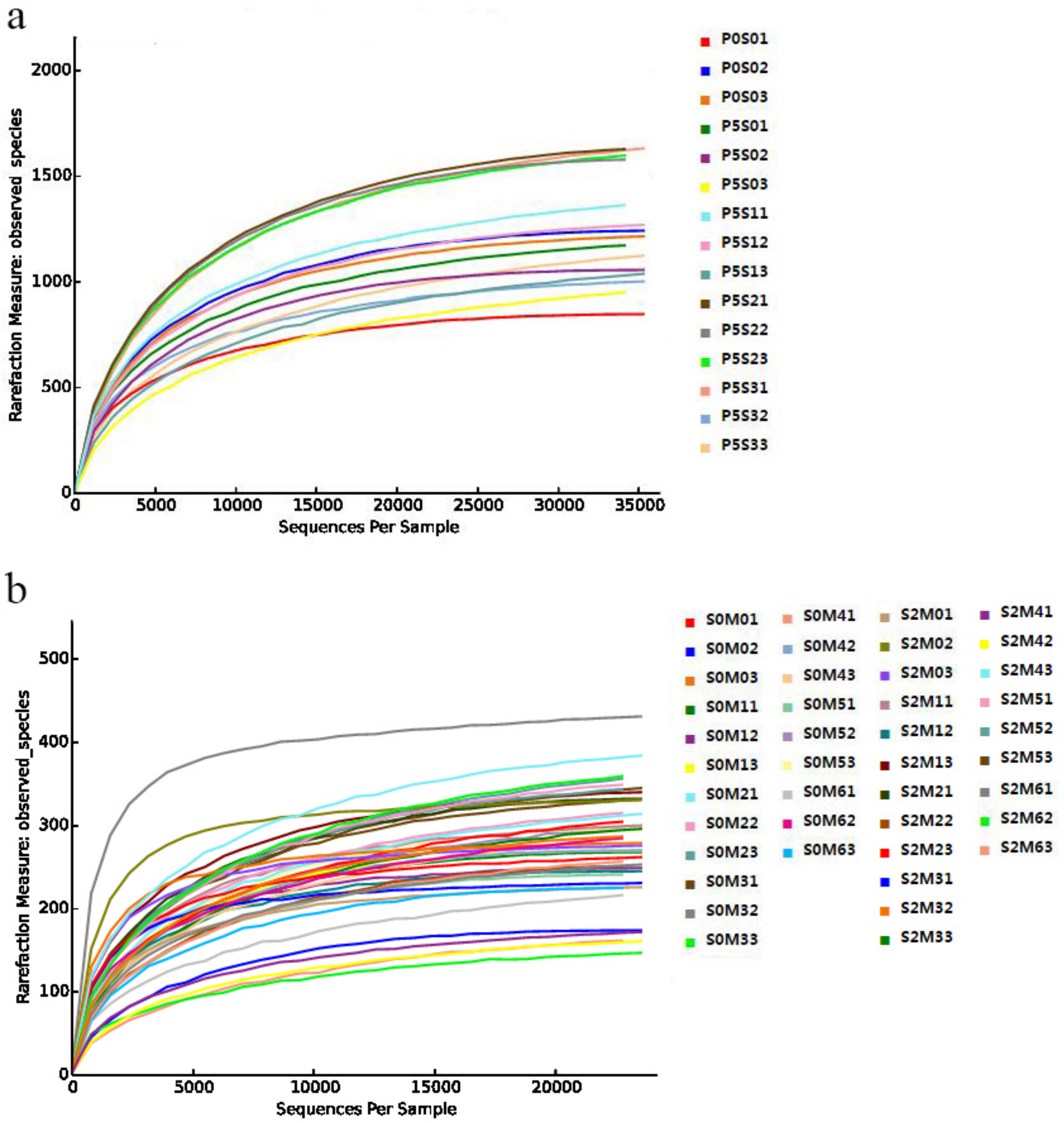

Supplementary Figure 1. Rarefaction curves for *norB*-type denitrifying bacterial (a) and fungal (b) OTU diversity among soil communities of different samples. Abbreviations: The seedlings treated with 0, 10, 100 and 1000 µmol/L sodium nitroprusside (SNP) were denoted S0, S1, S2, and S3, respectively. The seedlings that were irrigated with 0, 5 and 2000 µmol/L P nutrient solution were denoted as P0, P5, and P2000. Samples harvested at 0, 1, 2, 3, 4, 5, and 6 months after inoculation were denoted M0, M1, M2, M3, M4, M5, and M6, respectively.

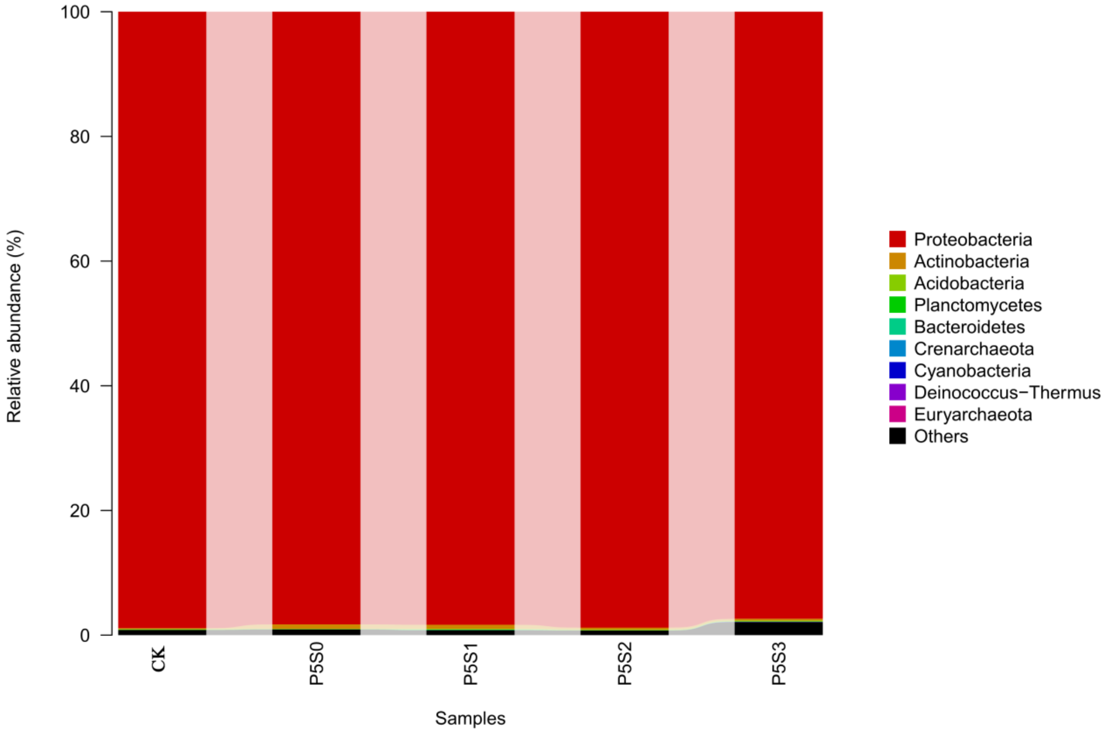

Supplementary Figure 2. Taxonomic composition of *norB*-type denitrifying bacterial communities at the phylum levels in rhizosphere soil of *C.illinoinensis* seedlings with different SNP treatments. All treatments were conducted with three replicates. CK, the rhizosphere soil of uninoculated *C.illinoinensis* seedlings which had 0 μmol/L SNP and phosphorus application. P_5S_0, P_5S_1, P_5S_2 and P_5S_3 were the rhizosphere soil samples of inoculated *C.illinoinensis* seedlings which respectively had 0 μmol/L, 10 μmol/L, 100 μmol/L and 1000 μmol/L SNP application under low P stress (5 μmol/L).

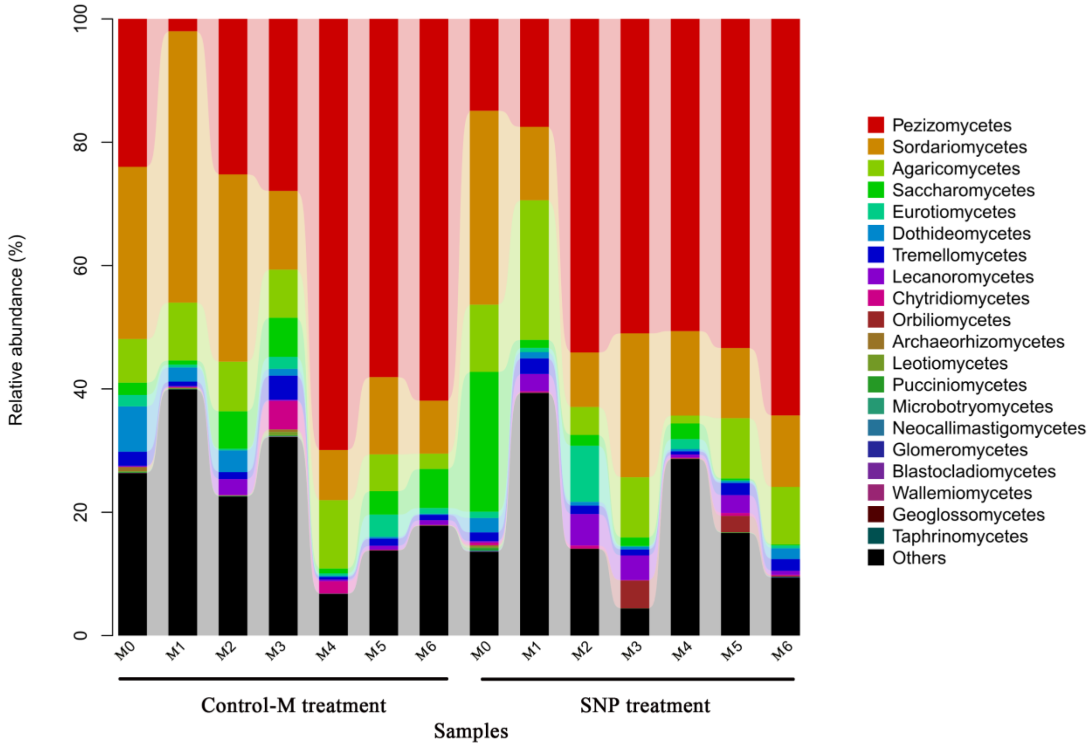

Supplementary Figure 3. Taxonomic composition of fungal communities at the class levels in rhizosphere soil of inoculated *C.illinoinensis* seedlings with or without SNP application on different growth months. All treatments were conducted with three replicates. Control-M treatment, the inoculated seedlings that didn't have 100 μmol/L SNP application. SNP treatment, the inoculated seedlings that had 100 μmol/L SNP application. M0, M1, M2, M3, M4, M5, and M6 respectively represent the rhizosphere soil harvested on the 0th, 1st, 2nd, 3rd, 4th, 5th, and 6th month after *T. indicum* inoculation.

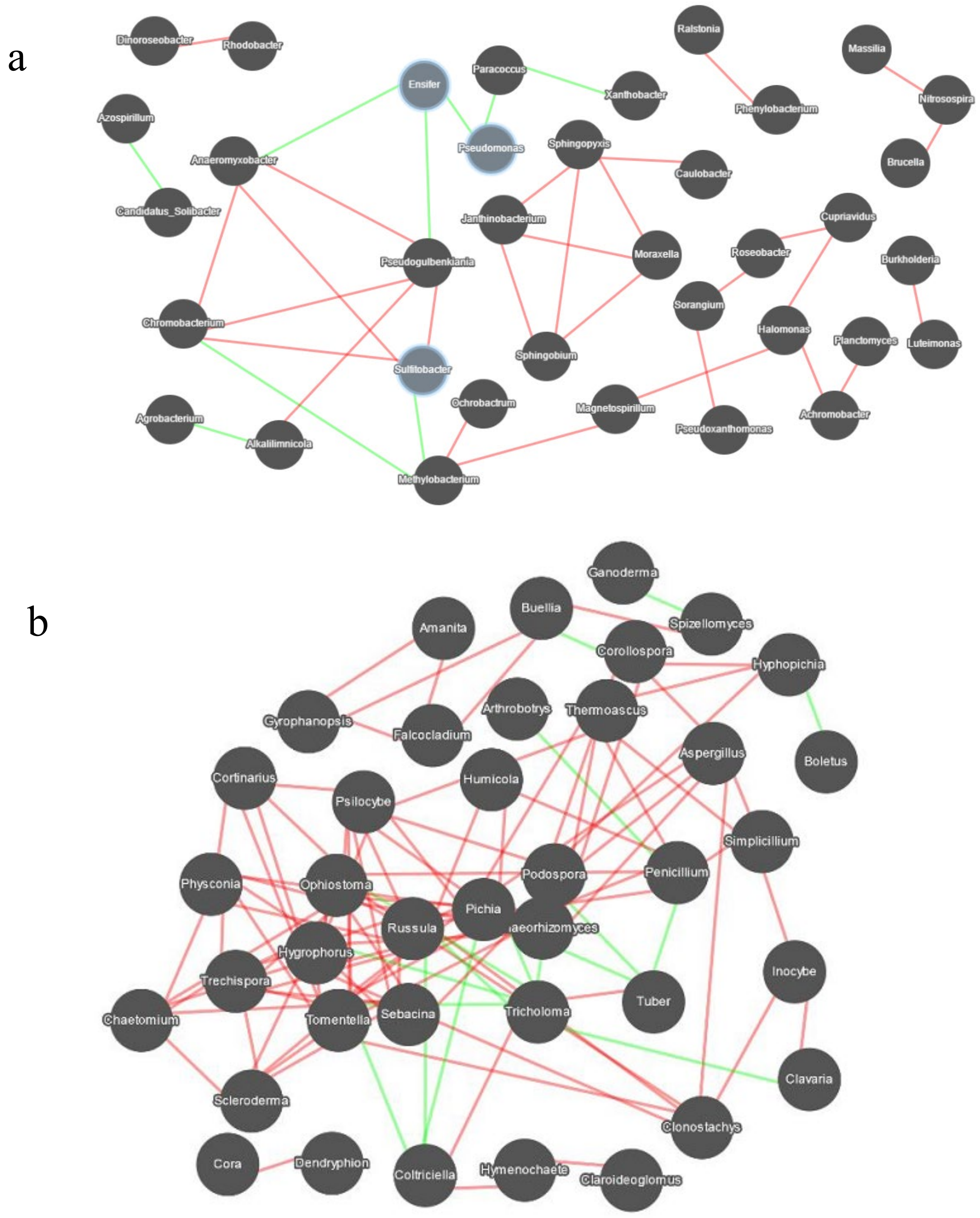

Supplementary Figure 4. Association network diagram of the dominant *norB*-type denitrifying bacterial genera (a) and fungal genera (b). The red and green edges between nodes indicate significant positive and negative correlations (Spearman's correlation coefficient (rho)>0.6 and P <0.01), respectively. A genus (node) displayed with more connections indicates its greater connectivity/interactions with other members in the network.

1.2 Supplementary Tables

Supplementary Table 1. The experimental design and sampling strategy in this study. (a) Exogenous NO treatment combined with P stress; (b) Exogenous NO treatment only.

Supplementary Table 1a

Sample ID/ Treatment	Cultivation substrate	Inoculation or not	P Stress	Exogenous NO (SNP)	Sample collection			Biological replicates
					Samples	Determination of indicators	Sampling time	
CK	Substrate I (nutrient-poor sands)	Not	0 μmol/L	0 μmol/L	a. Seedlings<br>b. Root system<br>c. Rhizospheres soil	a. Plant physiology<br>b. Colonization rate<br>c. *norB*-type denitrifying bacterial community	After 4 months from inoculation;	Three biological samples analyzed for each treatment
P_0S_0<br>P_0S_1<br>P_0S_2<br>P_0S_3		*T. indicum* inoculation	0 μmol/L	0 μmol/L<br>10 μmol/L<br>100 μmol/L<br>1000 μmol/L	a. Seedlings<br>b. Root system	a. Plant physiology<br>b. Colonization rate		
P_5S_0<br>P_5S_1<br>P_5S_2<br>P_5S_3			5 μmol/L	0 μmol/L<br>10 μmol/L<br>100 μmol/L<br>1000 μmol/L	a. Seedlings<br>b. Root system<br>c. Rhizospheres soil	a. Plant physiology<br>b. Colonization rate<br>c. *norB*-type denitrifying bacterial community		
$P_{2000}S_0$<br>$P_{2000}S_1$<br>$P_{2000}S_2$<br>$P_{2000}S_3$			2000 μmol/L	0 μmol/L<br>10 μmol/L<br>100 μmol/L<br>1000 μmol/L	a. Seedlings<br>b. Root system	a. Plant physiology<br>b. Colonization rate		

SNP: exogenous NO donor sodium nitroprusside; P: phosphorus

Supplementary Table 1b

Sample ID/ Treatment		Cultivation substrate	Inoculation or not	Exogenous NO	Sample collection			Biological replicates
					Samples	Determination of indicators	Sampling time	
Control-M	M0	Substrate II (organic soil, vermiculite and water at a volume ratio of 1:1:0.5)	*T. indicum* inoculation	0 μmol/L	a. Seedlings<br>b. Root system<br>c. Rhizospheres soil	a. Plant physiology<br>b. Fungal community	Every month after inoculation (0, 1, 2, 3, 4, 5, and 6 months after inoculation were denoted M0, M1, M2, M3, M4, M5, and M6)	Three biological samples analyzed for each treatment
	M1							
	M2							
	M3							
	M4							
	M5							
	M6					a. Colonization rate<br>b. Plant physiology<br>c. Fungal community		
SNP	M0			100 μmol/L		a. Plant physiology<br>b. Fungal community		
	M1							
	M2							
	M3							
	M4							
	M5							
	M6					a. Colonization rate<br>b. Plant physiology<br>c. Fungal community		

SNP: exogenous NO donor sodium nitroprusside;

Supplementary Table 2. The formula of Hoagland nutrient solution (except phosphorus).

Nutrient elements	Concentration (μmol/L)
N	16000
K	6000
Ca	4000
Mg	1000
S	1000
Fe	25.2-30.5
Mn	2.0
B	25
Zn	2.0
Cu	0.5
Mo	0.5
Cl	50

Supplementary Table 3. Spearman correlation coefficient between colonization rate and indicators of *norB*-type denitrifying bacterial communities

	Simpson	Shannon	Chao1	ACE
Colonazation rate	0.867**	0.900**	0.900**	0.900**

**Significant at $p < 0.01$
